# Supplementary material for: Utilizing the predictable binding kinetics of DNA-PAINT to denoise super-resolution images
Source: Nat Commun. 2026 Feb 5;17:2397. doi: 10.1038/s41467-026-69304-4 (PMC12982735; doi:10.1038/s41467-026-69304-4)
Supplement: Supplementary file 2 — Supplementary Information [file 41467_2026_69304_MOESM2_ESM.pdf]

Supplementary Figures

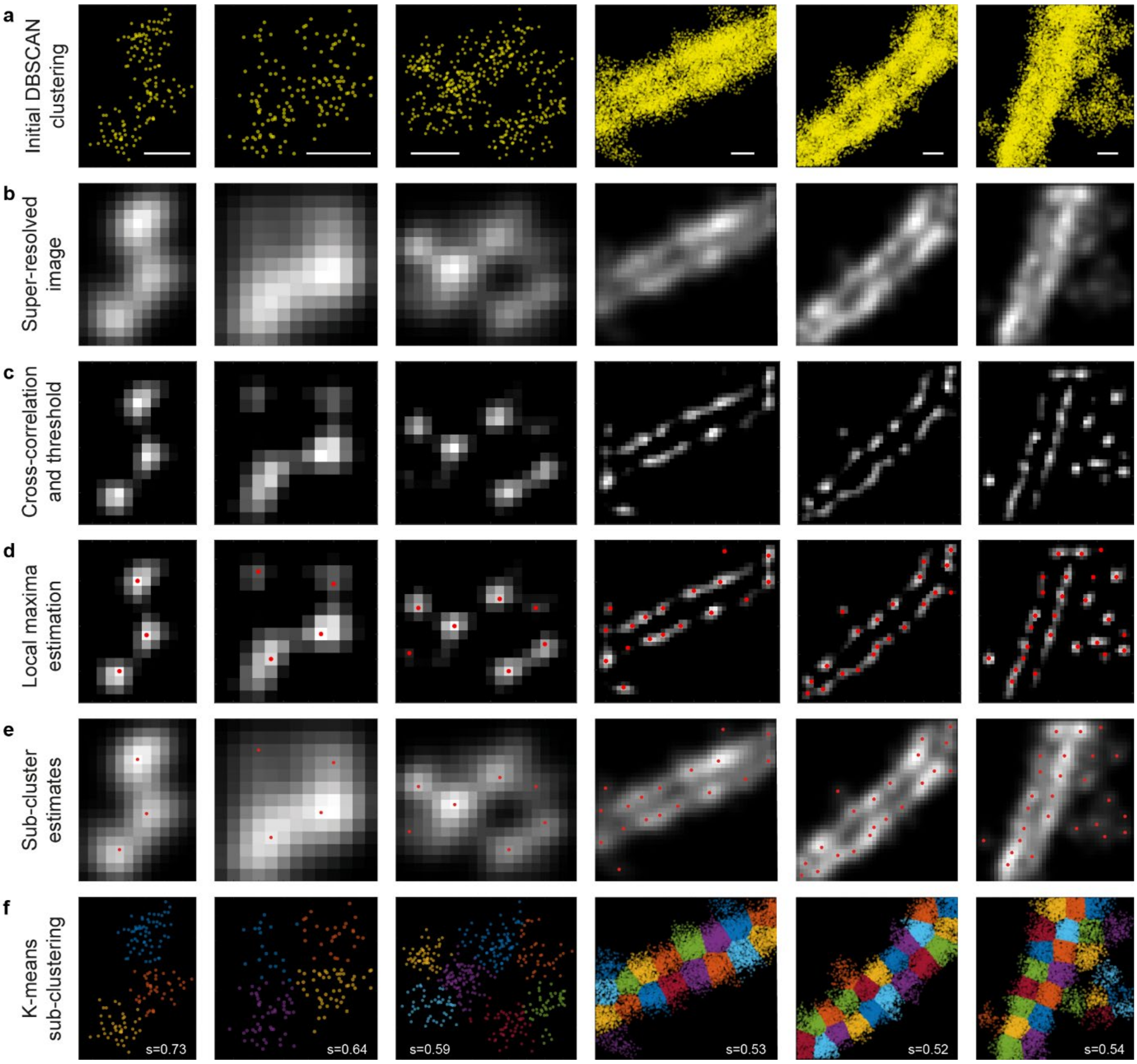

Supplementary Figure 1: Clustering of SMLM data. (**a**) Localizations clustered by the DBSCAN step are used to make a super-resolved image of each individual DBSCAN cluster shown in (**b**). (**c**) The normalized cross-correlation of each super-resolved DBSCAN cluster image shown in (b) and a 2D gaussian template image with a standard deviation that equals the localization precision is computed. This eliminates variations of the cross-correlation due to amplitude fluctuations in the DBSCAN cluster image and facilitates the application of a global threshold of 0.1. (**d**) Local maxima in the normalized cross-correlation shown in (c) are found using MATLAB's "imregionalmax" function and shown as red dots. (**e**) Local maxima found in (d) are used as estimates on the number and positions of the subclusters as indicated by the red dots. (**f**) Finally, the localizations in the DBSCAN cluster along with the estimates for the sub-clusters are fed into the k-means algorithm to partition the DBSCAN clusters to smaller localization-precision-sized clusters. s is the average silhouette score over all localizations in the DBSCAN cluster. Scale bar 50 nm

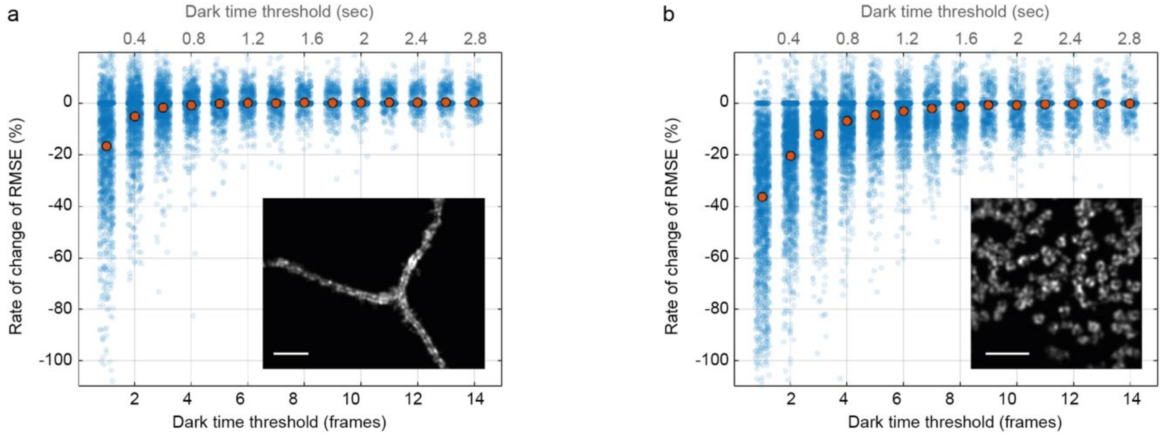

Supplementary Figure 2: Change in the RMSE of an exponential fit to the experimental CDF of the times between binding events as a function of the dark time threshold used to link the blinks. (**a**) Docking strand conjugated to the GFP-nanobody: Imaging E-Cadherin clusters along the borders of epithelial cells in stage 7 *Drosophila* egg chambers indicated that linking consecutives blinks separated by ~1 sec improves the exponential behaviour of the CDF. (**b**) Docking strand conjugated to the chloroalkane ligand: Imaging Nuclear Pore Complexes with the Nup160 expressing the HaloTag in nurse cells in stage 7 *Drosophila* egg chambers indicated that linking blinks separated by ~2 sec improves the exponential behaviour of the CDF. Scale bar 0.5 µm.

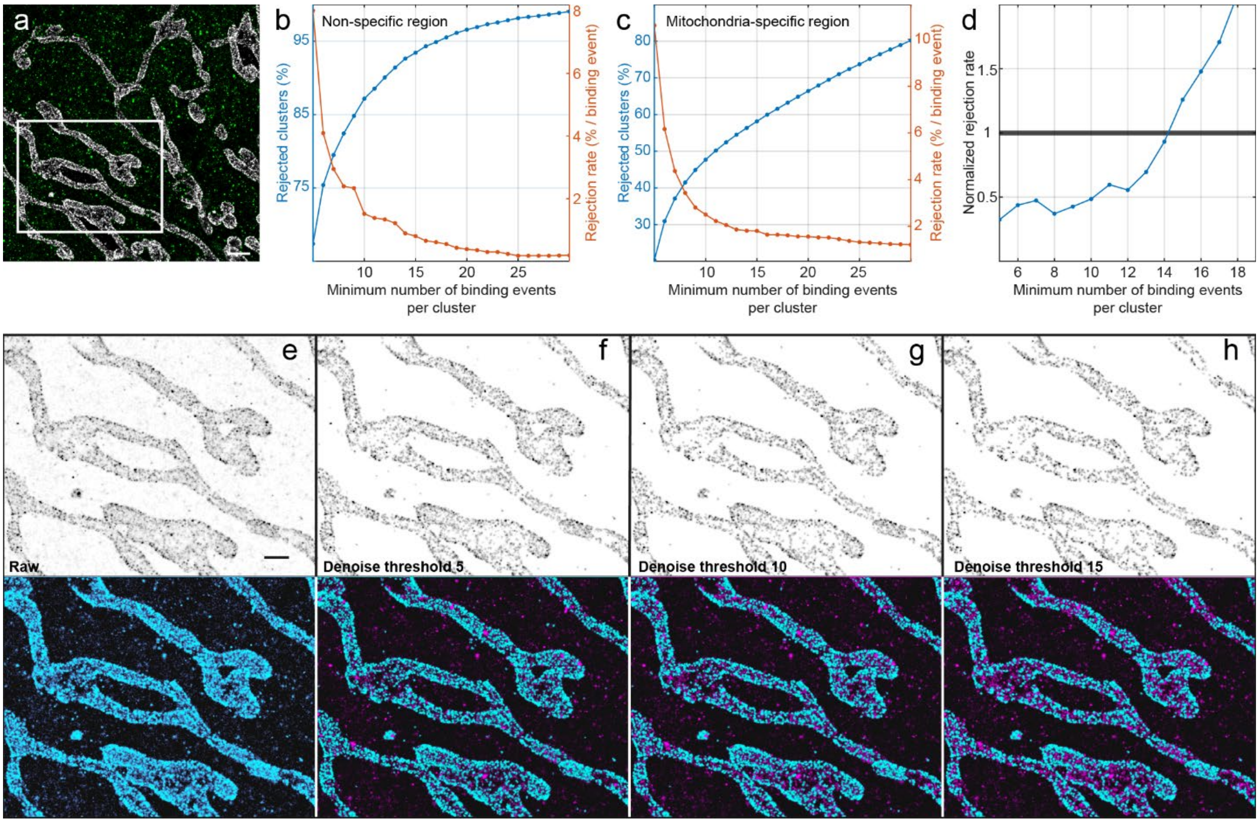

Supplementary Figure 3: Determining the threshold for the minimum number of binding events for mitochondria. (**a**) The characteristic shape of mitochondria facilitates the segmentation of the super-resolved image in mitochondria-specific region (shown in grey) and non-specific regions (shown in green). Scale bar 1 µm. Image is representative of 5 independent experiments. (**b**), (**c**) The fraction of rejected clusters (blue line) and the cluster rejection rate (orange line) are plotted as functions of the minimum number of binding events per cluster for the non-specific and the mitochondria-specific regions respectively. (**d**) The normalized rejection rate

of the mitochondria-specific region to the non-specific region as a function of the minimum number of binding events per cluster. (**e-h**) Raw and denoised images of mitochondria along with the corresponding scatter plots of the accepted (cyan) and rejected (magenta) localizations for a threshold of 5, 10 and 15 binding events per cluster. Scale bar 0.5 µm. A threshold of 5 binding events removes ~70% of the clusters in the areas outside the mitochondria. The remaining clusters in those areas are gradually removed with increasing denoising threshold and reach a level of ~94% for a denoising threshold of 15 binding events.

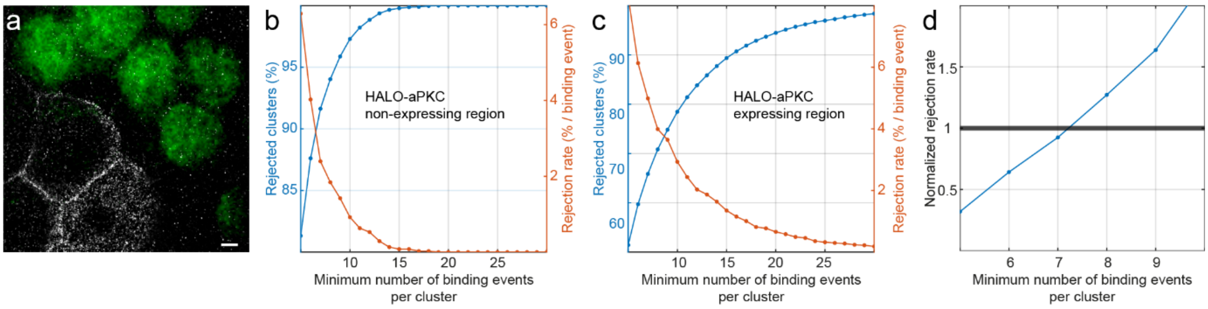

Supplementary Figure 4: Determining the minimum number of binding events for aPKC. (**a**) Overview image of a region of the follicle cell epithelium in a stage 7 *Drosophila* egg chamber containing cells that lack Halo-aPKC next to cells that are homozygous for Halo-aPKC. The cells that do not express Halo-aPKC are marked by nuclear GFP (green). Scale bar 1 µm. Image is representative of 3 independent experiments. (**b**), (**c**) The fraction of rejected clusters (blue line) and the cluster rejection rate (orange line) are plotted as functions of the minimum number of binding events per cluster for the expressing and non-expressing regions respectively. (**d**) Normalized rejection rate of the Halo-aPKC expressing region to the non-expressing region as a function of the minimum number of binding events per cluster.

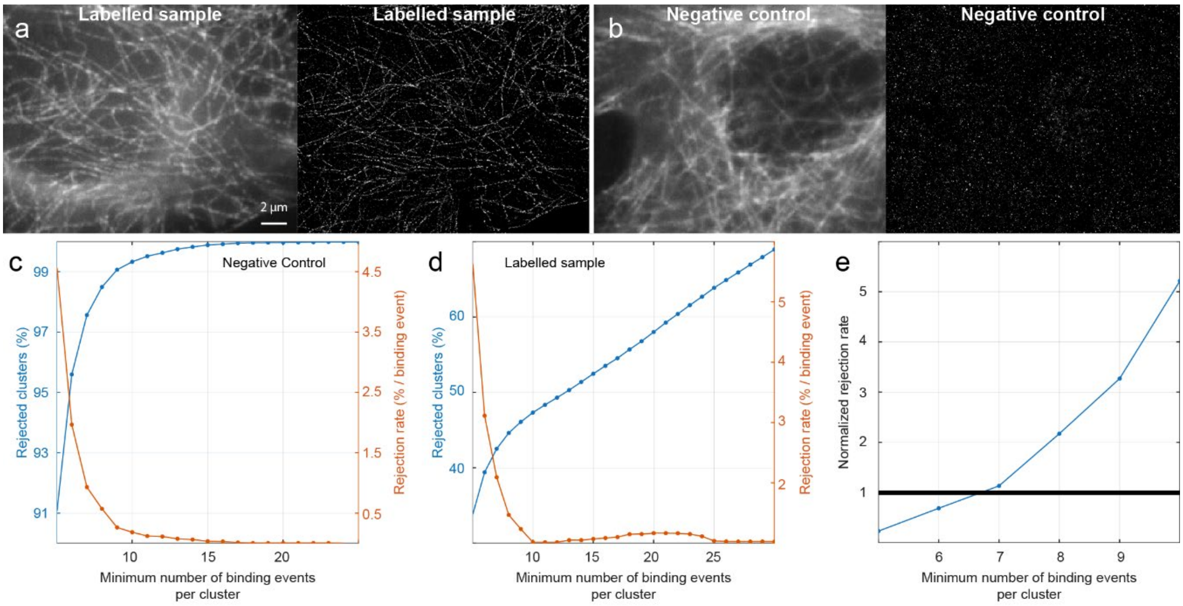

Supplementary Figure 5: Determining the threshold for the minimum number of binding events for microtubules based on a negative control sample. (**a**) Image of microtubules in a HeLa cell transfected with tubulin-GFP and labelled with a single domain antibody against GFP for DNA-PAINT imaging. The corresponding super-resolved image is shown next to the confocal spinning-disk GFP image. Images are representative of 4 independent experiments. (**b**) Image of a transfected cell used as a negative control for the denoising method. This sample was imaged and prepared under identical conditions to (a) except the nanobody labelling step was excluded. The corresponding super-resolved image lacks the characteristic filamentous structures of tubulin. Images are representative of 4 independent experiments (**c**), (**d**) The fraction of rejected

clusters (blue line) and the cluster rejection rate (orange line) are plotted as functions of the minimum number of binding events per cluster for the negative control and the labelled sample respectively. (**e**) The normalized rejection rate of the labelled sample to the negative control as a function of the minimum number of binding events per cluster.
